# Morphological description of the first protozoeal stage of the deep-sea shrimps *Aristeus antennatus* and *Gennadas elegans*, with a key

**DOI:** 10.1038/s41598-020-68044-9

**Published:** 2020-07-07

**Authors:** Marta Carreton, Antonina Dos Santos, Lígia Faria De Sousa, Guiomar Rotllant, Joan B. Company

**Affiliations:** 1 Institut de Ciències del Mar (ICM-CSIC), Passeig Marítim de la Barceloneta, 37-49, 08003 Barcelona, Spain; 2 Instituto Português Do Mar E da Atmosfera (IPMA), Av. Alfredo Magalhães Ramalho, 6, 1495-165 Algés, Portugal; 3 CIIMAR (Centro Interdisciplinar de Investigação Marinha E Ambiental), Terminal de Cruzeiros Do Porto de Leixões, Matosinhos, Portugal

**Keywords:** Biodiversity, Marine biology

## Abstract

Accurate information on commercial marine species larvae is key to fisheries science, as their correct identification is the first step towards studying the species’ connectivity patterns. In this study, we provide a complete morphological description of the first protozoeal stage of the valued deep-sea blue and red shrimp *Aristeus antennatus* and of the small mesopelagic shrimp *Gennadas elegans*. These two larval morphologies previously posed a risk of misidentification, thus hindering the study of *A. antennatus* larval ecology and dynamics in the context of fisheries science. Using specimens caught in the plankton at various locations in the Northwestern Mediterranean Sea and identification confirmed by molecular methods, the larvae of *A. antennatus* and *G. elegans* are distinguished from each other by the ornamentation of the antennula. A possible confusion in previous descriptions of Aristeidae larvae is addressed and a new key for the identification of Dendrobranchiata larvae provided.

## Introduction

Fisheries science depends on reliable and sufficient data about exploited species to build efficient strategies that ensure the durability of marine resources. One fundamental aspect of fisheries science is the study of species connectivity, as this information can shape the definition of stocks and set the range and scope of management instruments. Regardless of their adult habitat, many species have planktonic larvae. During this life phase, organisms are easily transported by currents; this plays a key role in terms of dispersal strongly influencing species’ connectivity and recruitment patterns^1–3^. For crustacean decapods, there is a well-documented body of knowledge about the larval stages of some exploited species^4–6^. However, this is not the case for deep-sea Dendrobranchiata, for which information is lacking despite the economic relevance in fisheries of some species. The scarcity of these larvae in plankton samples and the challenges of rearing these species in the laboratory are probably one of the main causes of the limited number of descriptive studies on the subject. As a result, observed data on deep-sea Dendrobranchiata larval abundance and distribution are scarce, and many of their larval stages are still undescribed^7^.

The deep-sea blue and red shrimp *Aristeus antennatus* (Risso 1816) is targeted by bottom trawlers in the entire Mediterranean Sea and the Northwestern coast of Africa. Its global catch reached 2,988 tonnes in 2016^8^ and in some areas like the Spanish Mediterranean coast, this species alone can represent up to 50% of fishermen associations’ yearly revenues^9,10^. Its adult biology has been thoroughly studied^11–13^, particularly in the Northwestern Mediterranean Sea, where it has been subject to a long-term co-management plan at a local scale^14^. The reproductive period of *A. antennatus* spans from May to September, with a peak in July and August, when females aggregate at the continental shelf break^15,16^. As for the mesopelagic shrimp *Gennadas elegans*, its distribution englobes the whole Atlantic Ocean and the Mediterranean Sea. It has no commercial interest but it is often caught accidentally by bottom trawlers targeting *A. antennatus*. The reproductive cycle of *G. elegans* has not yet been studied, but larvae of the species have been caught in the plankton all year round (e.g.^17^).

According to general knowledge about dendrobranchiate shrimps, the females spawn their eggs into the water column. The eggs then hatch into a nauplius, the first free-living larval phase which metamorphoses into a series of zoeal stages, often referred to as protozoea in their early stages and mysis during the late stages. The last mysis moults into a decapodid, which after a series of moults becomes a juvenile and begins searching for settlement in the adult habitat^18^. For *A. antennatus*, only 3 protozoeas and 2 mysis stages have been identified and described from plankton samples^20–22^. In 1955, Heldt^20^ described two larval series obtained from plankton samples in the Balearic Sea (Northwestern Mediterranean) and reared in laboratory conditions that she attributed to *Aristeus antennatus* and *Aristaeomorpha foliacea*. For *A. antennatus,* the publication presented the morphological description for the three protozoea stages and the first mysis stage; for *A. foliacea*, it described the last naupliar stage, the protozoea II and III and the first mysis stage. In particular, the first protozoea (PZ I) of *A. antennatus* was described from a single individual, whereas the PZ I of *A. foliacea* remained undescribed since, as mentioned by the author^20^, the single available specimen was lost. Occurrence of *A. antennatus* larvae in the plankton have been reportedly scarce^21,23–26^ until a recent study reported findings of all known larval stages of the species, with a particular high abundance of the PZ I^22^. For *G. elegans*, the only available description features only the PZ II and older stages^5^, while the description of the PZ I is included in a previous, more general study on the genus *Gennadas*^27^. Occurrence of *Gennadas* spp. PZ I has been widely reported in zooplankton studies (e.g.^21,27–29^).

Knowledge about Dendrobranchiata PZ I is particularly useful for fisheries science as this stage generally occurs from a few hours to a few days after hatching and can provide information on the spawning areas of the species^18^. Furthermore, information on larval behavior and distribution is essential to determine the connectivity patterns of commercial species and establish effective management strategies^30^. In this context, accurate identification of the larvae is key. The objective of this study was to accurately and comprehensively describe the first protozoeal stage of the deep-sea shrimps *A. antennatus* and *G. elegans*, to compare them in search for morphological distinguishing characters, and how the findings relate to previously available information.

## Results

**Figure 1:**
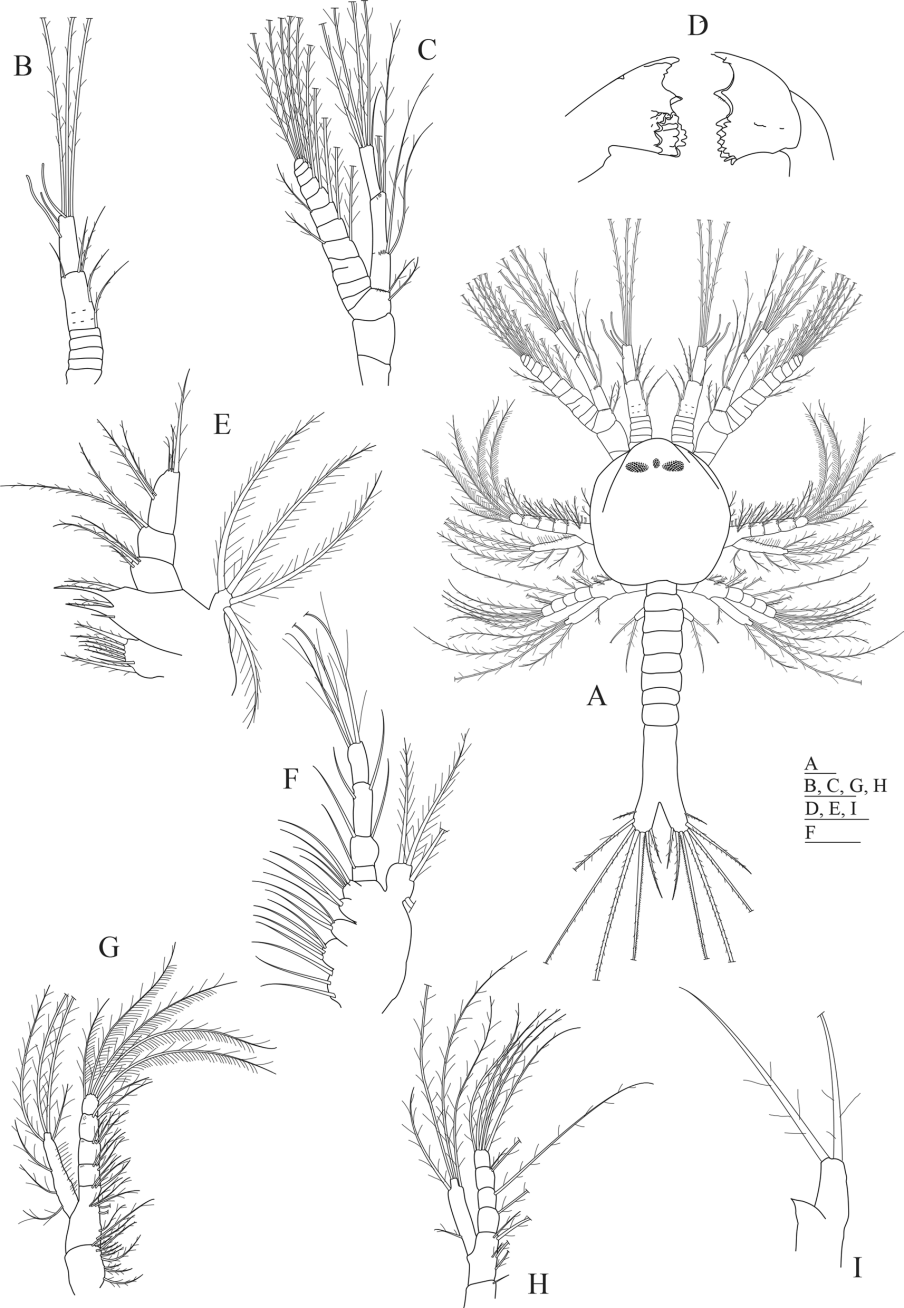
*Aristeus antennatus* first protozoea larva. (**A)** Dorsal view; (**B**) antennula; (**C**) antenna; (**D**) mandible; (**E**) maxillula; (**F**) maxilla; (**G**) first maxilliped; (**H**) second maxilliped; (**I**) third maxilliped. Drawn with GIMP software (v. 2.10.18, https://gimp.org).

**Figure 2:**
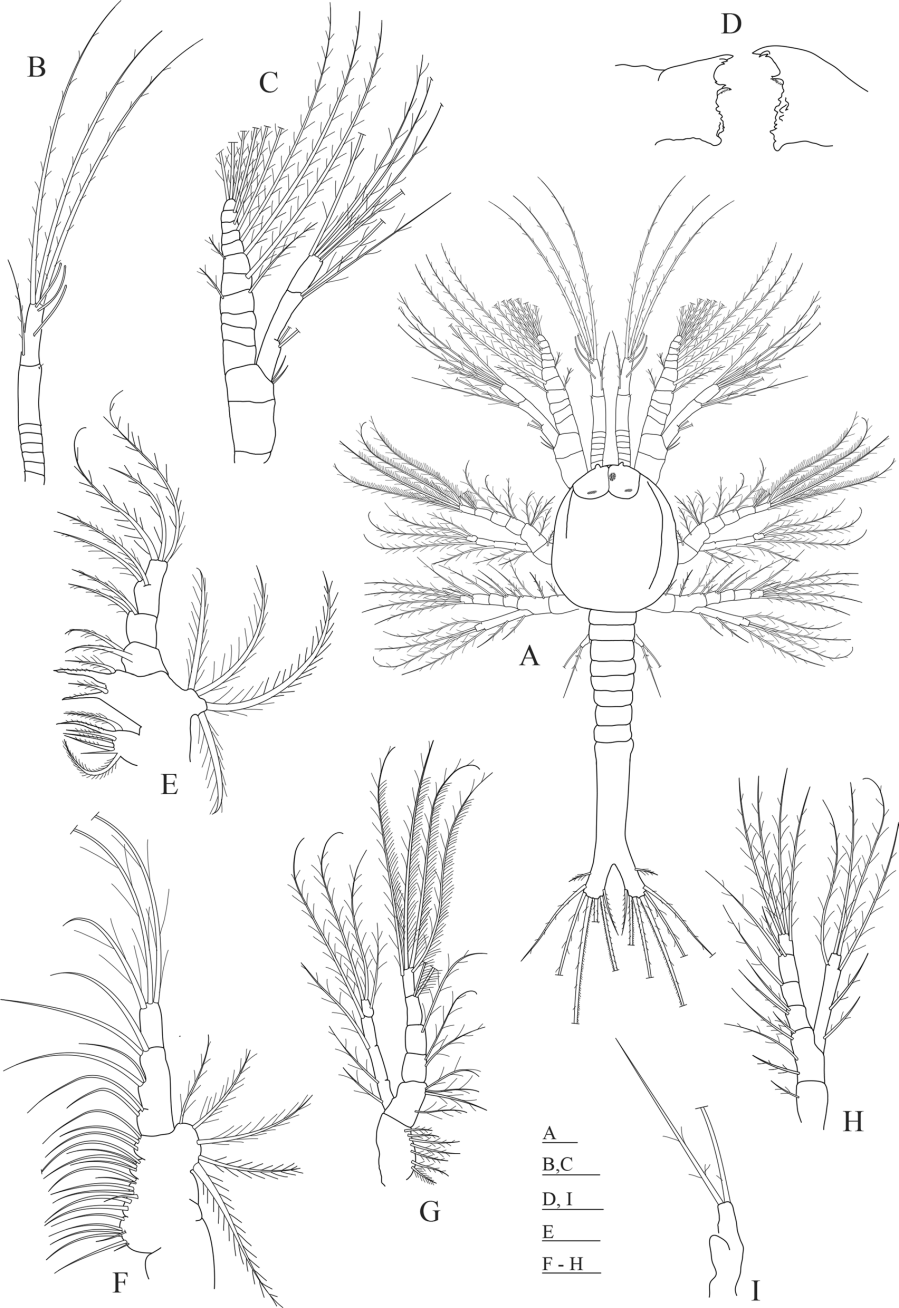
*Gennadas elegans* first protozoea larva. (**A**) Dorsal view; (**B**) antennula; (**C**) antenna; (**D**) mandible; (**E**) maxillula; (**F**) maxilla; (**G**) first maxilliped; (**H**) second maxilliped; (**I**) third maxilliped. Drawn with GIMP software (v. 2.10.18, https://gimp.org).

**Figure 3:**
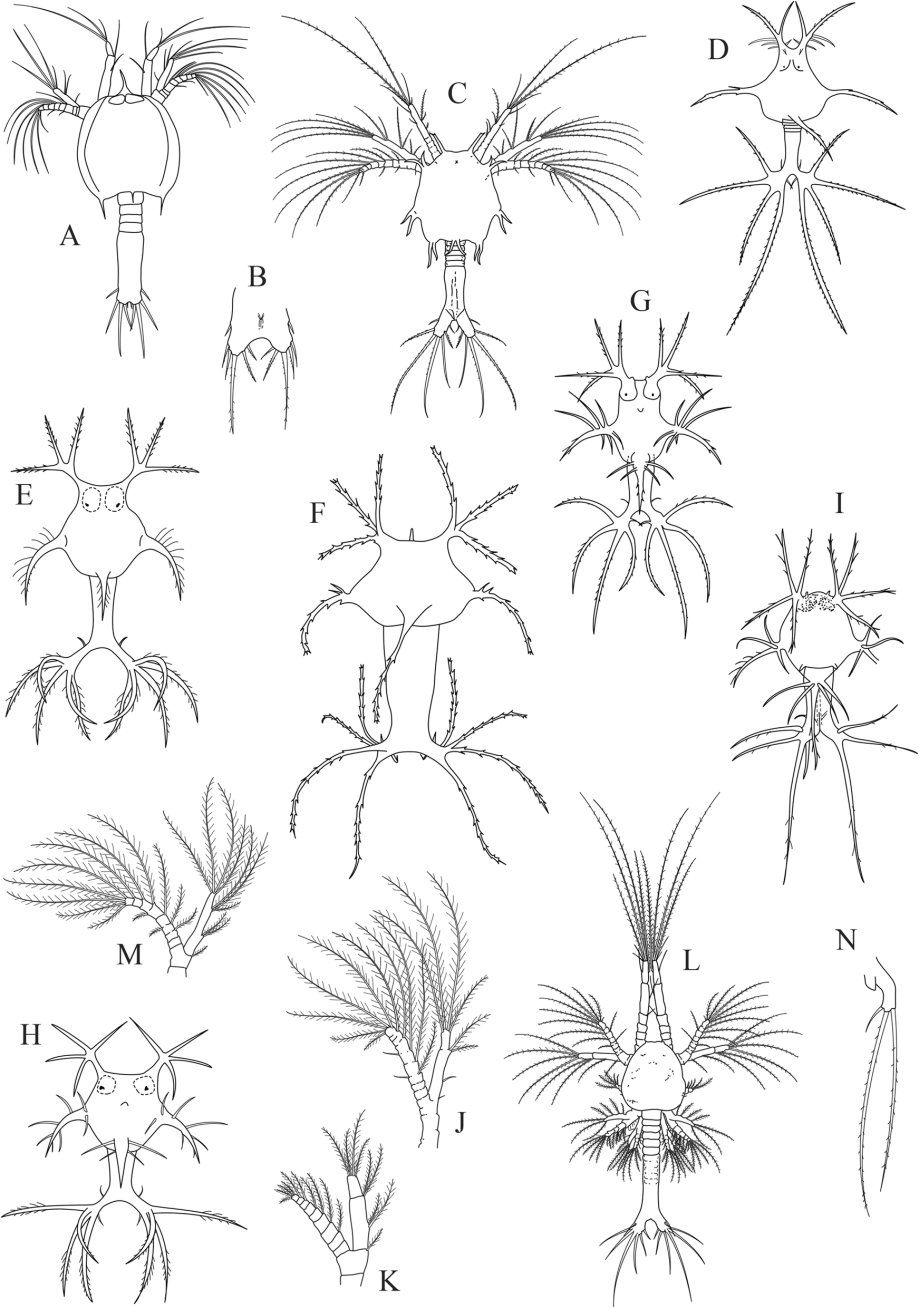
Drawings of known protozoea I larvae of Dendobranchiata species. (**A**) Dorsal view of *Lucifer penicillifer*; (**B**) telson of *Petalidium* sp.; (**C**) dorsal view of *Solenocera membranacea*; (**D**) dorsal view of *Parasergestes vigilax*; (**E**) dorsal view of *Sergestes atlanticus*; (**F**) dorsal view of *Eusergestes arcticus*; (**G**) dorsal view of *Deosergestes corniculum*; (**H**) dorsal view of *Sergia remipes*; (**I**) dorsal view of *Deosergestes henseni*; (**J**) antenna of *Penaeus kerathurus*; (**K**) antenna of *Penaeopsis rectacuta*; (**L**) dorsal view of *Sicyonia carinata*; (**M**) antenna of *Parapenaeus longirostris*; (**N**) third maxilliped of *Aristaeomorpha foliacea*. All figures redrawn with GIMP software, (v. 2.10.18, https://gimp.org) from: A. ^36^; B and I. ^27^; C. ^37^; D, E, G and H. ^38^; F. ^39^; J, L and M. ^5^; K. ^40^; N. ^20^. Drawings not to scale.

The main differential morphological characters between the first protozoea stage of the two species are summarized in Table 1. Also, we propose an identification key to distinguish the first protozoeal stage of Dendrobranchiata larvae of species occurring in the Northeastern Atlantic ocean and Mediterranean Sea, gathering information from our own observations and from available literature^31–38^. The general body morphology description of the Dendrobranchiata first protozoea stage can be found in some recent references^18,19^. The first protozoea (PZ I) of Dendrobranchiata larvae has a carapace covering part of the cephalotorax, followed by an unsegmented pleon and finishing in a large bilobed telson (Figs. 1A, 2A, 3A, C–I, L). The carapace is unarmed in most of the Penaeidae (Figs. 1A, 2A, 3L) but the Solenoceridae (Fig. 3C), the Luciferidae (Fig. 3A) and the Sergestidae (Fig. 3D–I) possess dorsal and lateral spines or processes. The compound eyes are covered by the carapace (e.g. Fig. 3E), and the naupliar eye is still visible (Fig. 3C). These larvae have two pairs of antennae in the anterior part of the carapace: the first pair (antennula) is uniramous and the second one (antenna) is biramous. In the antennae (e.g. Fig. 3K, J, M), the exopod is composed by a long plumose outer ramus with several ringlets throughout its length, and the endopod is the inner ramus. The mouth appendices are composed by a pair of mandibles, with incisor and molar processes, and two pairs of maxillae. The larvae also present 2 pairs of biramous maxillipeds where the outer ramus is the exopod and the inner ramus is the endopod. The third pair of the maxilliped, when present, is still rudimentary.

**Table 1:** Summary of most relevant differential morphological characters between *Aristeus antennatus* and *Gennadas elegans* protozoea I larvae and the previous morphological description of the same larval stage attributed to *A. antennatus*. a: aesthetascs, s: setae.

Features	*Gennadas elegans* (this study)	*Aristeus antennatus* (this study)	*Aristaeomorpha foliacea* as *Aristeus antennatus* (Heldt, 1955)
Total length (mm)	0.86–1.22	1.12–1.25	1.55
Carapace length (mm)	0.33–0.44	0.37–0.49	Not available
Naupliar eye	Present	Present	Present
Eyes	Compound eyes feebly dark	Compound eyes well formed and dark	Compound eyes well formed and dark
Pereion, frontal organs	Present	Present	Present
Antennula, number of setae (s) and aesthetascs (a) on somites	0, 1 s, 1 s + 3a + 3 s	1 s,4 s, 3a + 3 s	1 s,4 s,2a + 3 s
Antenna, protopod and endopod setal formula	2 + 2 + 2	2 + 2 + 2	Not available
Maxillula	Basial endite: 4 s; Endopod: 2 s, 2 s, 2 s + 3 s	Basial endite: 5 s; Endopod: 3 s, 2 s, 2 s + 3 s	Basial endite: 4 s; Endopod: 2 s, 2 s,1 s,3 s
Maxilla	Basial endite: 5 s + 4 s + 3 s, plumose		
	Endopod: 2-segmented, 2 s + 2 s + 2 s, 3 s	Endopod: 1 s, 2 s, 2 s, 3 s	Endopod: 2 s, 2 s, 2 s, 3 s
	Exopod: 5 s, long plumose	Exopod: 4 s	Exopod: 5 s
First maxilliped	Endopod: 4-segmented, 2 s, 1 s, 2 s, 4 s	Endopod: 3 s, 3 s, 2 s, 5 s	Endopod: 2 s, 2 s, 3 s, 5 s
	Exopod: 2-segmented, 1 + 1 + 1 + 1 s, 3 s, 2 plumose setae on distal margin	Exopod: 1 + 4 s	Exopod: 1 + 1 + 1 + 4 s
Second maxilliped	Endopod: 2 s, 1 s, 2 s, 4 s Exopod: 1 + 1 + 4 s	Endopod: 1 s, 1 s, 2 s, 5 s Exopod: 1 + 4 s	Endopod: 2 s, 2 s, 1 s, 1 s, 5 s Exopod: 1 + 1 + 4 s
Third maxilliped	Endopod: as bud Exopod: 2 long plumose setae	Endopod: as bud Exopod: 2 long plumose setae	Endopod: as bud Exopod: 3 s (1 short, 2 long)

### Morphological description of Protozoea I of *Aristeus antennatus* (Fig. 1)

Size: TL (total length) = 1.12–1.25 mm; CL (carapace length) = 0.37–0.49 mm; N (number of protozoea examined) = 13.

Carapace (Fig. 1A): carapace almost rounded, longer than wider, reaching the level of the second maxilliped, with frontal organs visible at the anterior part; naupliar eye present flanked by a pair of compound eyes that are already visible through the carapace; 6 thoracic somites visible.

Antennula (Fig. 1A,B): first paired uniramous appendage in the cephalotorax, consisting of 3 articles: proximal article subdivided in 5 ringlets, bearing 1 short serrulate seta on the posterior end; second article with 1 positioned at mid-length of article and 3 serrulate setae distally; distal article with 3 aesthetascs subterminally and 3 long sparsely plumose setae on the posterior end.

Antenna (Fig. 1A,C): second paired biramous appendage in the cephalotorax, longer than antennula, consisting of a peduncle, an endopod and an exopod. Peduncle 3-segmented with 1 + 1 sparsely plumose setae on distal segment; endopod 2-segmented with 2 + 2 lateral plumose setae in proximal segment and 4 long plumose and 1 simple setae in the posterior segment; exopod with 11 ringlets, 3rd ringlet with a transversal incomplete separation, ringlets 4th to 11th each with a long plumose setae along inner margin and two more long plumose setae on the terminal position of the 11th ringlet, 4th and 6th ringlets each with an additional plumose setae on outer margin.

Mandible (Fig. 1D): the first paired appendage following the mouth placed in the ventral side of the cephalotorax, with distinct incisor and molar processes; incisive part with 2 + 2 teeth (the one placed near the molar part is short serrated); molar part with numerous small conate and uncinate teeth; without palp.

Maxillula (Fig. 1E): the second paired appendage following the mouth placed in the ventral side of the cephalotorax, divided in coxa, basipod, endopod and exopod. Coxa with 7 (1 simple and 6 papposerrate) setae; basipod with 5 (1 simple and 4 cuspidate) setae; endopod 3-segmented with 2 sparsely plumose and 1 small simple setae on the proximal segment, 2 sparsely plumose setae on second segment and 2 + 3 sparsely plumose setae on distal segment; exopod as a small knob-like structure with 4 long plumose setae.

Maxilla (Fig. 1F): the third paired appendage following the mouth placed in the ventral side of the cephalotorax, composed of coxa, basipod, endopod and scaphognathite. Coxal endite bilobed with 3 + 4 plumose setae; basial endite trilobed with 3 + 2 + 4 plumose setae; endopod 4-segmented bearing 1,1 + 1,1 + 1,3 long plumose setae; scaphognathite with 4 marginal long plumose setae.

First maxilliped (Fig. 1A,G): biramous paired appendage placed in the penultimate thoracic somite covered by the carapace, consisting of a protopod, an endopod and an exopod. Protopod 2-segmented (coxa and basipod), proximal coxal portion with 10 papposerrate setae; distal basial portion with 2 + 5 papposerrate setae along margin and 3 papposerrate seta on distal end; endopod 4-segmented with 3, 3, 2, 5 papposerrate setae; exopod unsegmented with 3 long and 4 plumose setae on distal margin.

Second maxilliped (Fig. 1A,H): biramous paired appendage placed in the last thoracic somite covered by the carapace, divided in coxa, basipod, endopod and exopod. Coxa with 1 seta; basipod with 1 + 2 + 2 papposerrate setae; endopod 4-segmented with 1, 1, 2, 4 papposerrate setae; exopod unsegmented with 1 + 4 long plumose setae.

Third maxilliped (Fig. 1A,I): biramous paired appendage placed in the first thoracic somite not covered by the carapace, consisting of an endopod and an exopod. Endopod represented by a small bud tapered at the end; exopod unsegmented with 2 long plumose setae distally.

Pereiopods: absent.

Pleon (Fig. 1A): pleomeres not completely differentiated, united with the telson and unarmed.

Pleopods: absent.

Uropods: absent.

Telson (Fig. 1A): broadly bifurcate with two distinct branches, each branch with 7 long plumose spines except the outermost one that is simple.

### Morphological description of Protozoea I of *Gennadas elegans* (Fig. 2)

Size: TL = 0.86–1.22 mm; CL = 0.33–0.44 mm; N = 9.

Carapace (Fig. 2A): carapace almost rounded, longer than wider, reaching the level of the second maxilliped, with frontal organs visible at the anterior part; naupliar eye present flanked by a pair of compound eyes that are already visible through the carapace; 6 thoracic somites visible and not covered by the carapace.

Antennula (Fig. 2A,B): first paired uniramous appendage in the cephalotorax, consisting of 3 articles: proximal article subdivided in 5 ringlets; second article with 1 very small simple spine distally; distal article with 3 aesthetascs and 1 sparsely plumose setae subterminally and 3 long sparsely plumose setae on the distal end.

Antenna (Fig. 2A,C): second paired biramous appendage in the cephalotorax, longer than antennula, consisting of a peduncle, an endopod and an exopod. Peduncle 3-segmented with 2 sparsely plumose setae on distal segment; endopod 2-segmented with 2 + 2 lateral plumose setae in proximal segment and 4 long + 1 short plumose setae in the distal somite; exopod with 11 ringlets, ringlets 4th to 11th each with a long plumose setae along inner margin and two more long plumose setae on the terminal position of the 11th ringlet, 4th and 6th ringlet each with an additional plumose setae on outer margin.

Mandible (Fig. 2D): the first paired appendage following the mouth placed in the ventral side of the cephalotorax, with distinct incisor and molar processes; incisive part with 3 (one minute) + 2 (the one placed near the molar part is serrated) teeth; molar part with numerous small connate and uncinated teeth; without palp.

Maxillula (Fig. 2E): the second paired appendage following the mouth placed in the ventral side of the cephalotorax, divided in coxa, basipod, endopod and exopod. Coxa with 7 (1 simple and 6 papposerrate) setae; basipod with 4 (2 cuspidate and 2 papposerrate) setae; endopod 3-segmented with 2 sparsely plumose setae on the proximal segment, 2 sparsely plumose setae on second somite and 2 + 3 sparsely plumose setae on distal segment; exopod as a small knob-like structure with 4 long plumose setae.

Maxilla (Fig. 2F): the third paired appendage following the mouth placed in the ventral side of the cephalotorax, composed of coxa, basipod, endopod and scaphognathite. Coxal endite bilobed with 7 (1 small simple) + 2 plumose setae; basial endite trilobed with 5 + 4 + 3 plumose setae; endopod 2-segmented bearing 2 + 2 + 2, 3 long plumose setae; scaphognathite with 5 marginal long plumose setae.

First maxilliped (Fig. 2A,G): biramous paired appendage placed in the penultimate thoracic somite covered by the carapace, consisting of a protopod, an endopod and an exopod. Protopod 2-segmented (coxa and basipod), proximal coxal portion with 7 papposerrate setae; distal basial portion with 1 + 3 papposerrate setae; endopod 4-segmented with 2, 1, 2, 4 papposerrate setae; exopod 2-segmented with 1 + 1 + 1 + 2 setae along margin of proximal segment and 2 plumose setae on distal margin of terminal segment.

Second maxilliped (Fig. 2A,H): biramous paired appendage placed in the last thoracic somite covered by the carapace, divided in coxa, basipod, endopod and exopod. Coxa with 1 papposerrate seta; basipod with 1 + 2 + 2 papposerrate setae; endopod 4-segmented with 2, 1, 2, 4 papposerrate setae; exopod unsegmented with 1 + 1 + 2 + 2 long plumose setae.

Third maxilliped (Fig. 2A,I): biramous paired appendage placed in the first thoracic somite not covered by the carapace, consisting of an endopod and an exopod. Endopod represented by a small bud rounded at the end; exopod unsegmented with 2 long plumose setae distally.

Pereiopods: absent.

Pleon (Fig. 2A): 2 pleomeres differentiated, all the others united with the telson and unarmed.

Pleopods: absent.

Uropods: absent.

Telson (Fig. 2A): broadly bifurcate with two distinct branches, each branch with 7 long plumose spines.

### Identification key for the first protozoeal stage of Dendrobranchiata larvae of the Northeastern Atlantic and Mediterranean Sea

**Table:** 

1	Rostrum present (Fig. 3A)	2
	Rostrum absent (Fig. 3C–I, L)	3
2	Telson with 5 pairs of spines (Fig. 3A)	*Lucifer* and *Belzebub*
	Telson with 6 pairs of spines (Fig. 3B)	*Petalidium*
3	Pereion margin with spines or processes (Fig. 3C–I)	4
	Pereion margin smooth (Fig. 3L)	10
4	Pereion octagonal with a pair of robust spines at each vertice (Fig. 3C)	*Solenocera membranacea*
	Pereion with anterior, lateral and posterior processes (Fig. 3D–I)	5
5	Pereion anterior process with 3 branches (Fig. 3D–F)	6
	Pereion anterior process with 4 branches (Fig. 3G–I)	8
6	Median branch of the anterior process of pereion with denticles only (Fig. 3D)	*Parasergestes vigilax*
	All branches of anterior pereion process with denticles (Fig. 3E, F)	*7*
7	Telson branches long and narrow, length more than 3 times the width (Fig. 3E)	*Sergestes atlanticus*
	Telson branches short, length only slightly greater than width (Fig. 3F)	*Eusergestes arcticus*
8	Posterior process of pereion swollen at base (Fig. 3G)	*Deosergestes corniculum*
	Posterior process of pereion not swollen at base	*9*
9	Lateral process with 7 long spines at the base (Fig. 3H)	*Sergia remipes*
	Lateral process with 3 long spines at the base (Fig. 3I)	*Deosergestes henseni*
10	Setal formula of antennal protopod and endopod is 1,1,2, third maxilliped absent (Fig. 3J)	*Penaeus (Melicertus) kerathurus*
	Setal formula of antennal protopod and endopod is 1,2,2 (Fig. 3K)	*Penaeopsis*
	Setal formula of antennal protopod and endopod is 1,2,3 (Fig. 3M)	11
	Setal formula of antennal protopod and endopod is 2,2,2 (Figs. 1C, 2C)	12
11	Length of antennula 2 × longer than antenna (Fig. 3L)	*Sicyonia carinata*
	Length of antennula approximately equal to that of antenna	*Parapenaeus longirostris*
12	Exopod of the third maxilliped with 3 setae (Fig. 3N)	*Aristaeomorpha foliacea*
	Exopod of the third maxilliped with 2 setae (Figs. 1I, 2I)	*13*
13	Setal formula of antennula is 0,1,4 (Fig. 2B)	*Gennadas elegans*
	Setal formula of antennula is 1,4,3 (Fig. 1B)	*Aristeus antennatus*

### Discussion

Although morphologically quite similar in most of their characters, the first protozoeal stages of *A. antennatus* and *G. elegans* bear some differences that will allow to distinguish them, as shown in Table 1 and in the identification key proposed. The first protozoea of *A. antennatus* presents 1, 4, 3 setae along the segments of the antennula, whereas in the case of *G. elegans*, the setal formula is 0, 1, 4. These characters are relatively easy to observe at the stereomicroscope, in most cases without the need of dissecting the specimens, and should provide an easy guide to differentiating the first protozoea of these two species.

The identification and morphological description of the larval series of *A. antennatus* found in the plankton off the Balearic archipelago by Heldt in 1955^20^ has proven to be fundamentally correct, as the descriptions of the rest of known stages of the species—PZ II, PZ III and mysis I—have been recently confirmed^22^. However, when comparing the *A. antennatus* PZ I from the present study with the one described by Heldt^20^, we found differences in the size of the larvae—the sole specimen in the cited study measured 1.55 mm, whereas in the present study the average total length is 1.2 mm. Moreover, we found differences between the two studies in the number of aesthetascs on the antennula, and in the number of setae on the exopod of the third maxilliped. While the possibility of an error can never be excluded, Heldt’s meticulous work and thorough descriptions in all her publications on Penaeid larvae make it unlikely that she would draw and describe a morphological character that she did not observe. We here expose our considerations about this contradiction.

First, Heldt’s study refers that one single specimen of first protozoea stage was caught for each of the studied species, *A. antennatus* and *A. foliacea*, but that the latter was apparently lost during manipulation and could not be described. Second, as seen in Table 1, the total length of the *A. antennatus* PZ I specimen measured by Heldt is 1.55 mm, while the next stage, PZ II, measured 1.50–2.03 mm^20^: this would mean that the PZ II was smaller than its previous stage. Variability in total length of these larvae has not been studied and might allow for such values, but Carreton et al.^22^ found an average total length of only 1.2 mm (± 0.05) for the PZ I. On the other hand, the PZ II of *A. foliacea* examined by Heldt measured 1.9 mm^20^ which is more in agreement with the length of the PZ I larva described as *A. antennatus*. Finally, Heldt’s description of *A. antennatus* PZ I accounts for 3 setae on the exopod of the third maxilliped (mxp3), whereas in our findings, all individuals presented only 2 setae. Furthermore, it seems that, in Heldt’s description, *A. foliacea* PZ II larvae present more developed characters than *A. antennatus* PZ II, as the mxp3 is described in *A. foliacea* with 3 setae on the exopod and 2 on the endopod, while in the case of *A. antennatus,* it only presents setae on the exopod. It would then be possible that, in the case of the PZ I, the more setose (3-setae) third maxilliped belongs to *A. foliacea* and the less setose (2-setae) one belongs to *A. antennatus*. For these reasons, we conclude that Heldt’s description of *A. antennatus* PZ I is probably that of *A. foliacea*. The PZ I of *A. antennatus* would then have remained undescribed until now.

The present study provides the first detailed morphological description of the protozoea I larvae of *A. antennatus* and *G. elegans* according to modern standards, made from plankton samples after identification being confirmed with molecular analysis. The protozoea I larvae of the two studied species can be morphologically distinguished from one another mainly by the setation of the antennula. An identification key is provided allowing for the morphological identification of all first protozoea larvae of Dendrobranchiata for the Mediterranean Sea and Northeast Atlantic Ocean known today.

In a context where fisheries science is increasingly drawing on marine connectivity to design regional-scale management strategies for commercial species, larval distribution studies are one of the first stepping stones to effective planning, as they broaden the knowledge on species dispersal patterns. It is then essential to ensure a correct identification of these larvae, and morphological characters provide accurate, at-hand information even when molecular methods are not applicable. Our results set a starting point for *A. antennatus* connectivity studies in the frame of fisheries management, and we are confident that the identification key provided will make classification of the featured early larval stages accessible to both taxonomers in the field and non-specialists.

## Method

### Specimen collection

For *A. antennatus* larvae, the sampling was carried out in August 2016 in various locations off the Spanish Mediterranean coast (Table 2). We used a neuston sledge with a 300-µm mesh net between 0.5 and 1 m depth over bottoms of 123 to 1626 m. For *G. elegans* larvae, we sampled 3 stations off the Catalan coast in February 2017 (Table 2). The selection of this second sampling interval outside of the reproductive period of *A. antennatus* was deliberate in order to avoid collecting a mix of the two species. We used a 60-cm diameter bongo with a 300-µm mesh net in oblique tows between 500 m depth and the surface, over bottoms of 1,952 and 1,790 m. All PZ I larvae from both samplings were sorted and identified at the stereomicroscope using the available keys and descriptions^20,21,31^ and stored individually in 96% ethanol.

**Table 2:** Information on the larvae observed.

Date	Lon (°E)	Lat (°N)	Gear	Mesh size (µm)	Sampling depth (m)	Bottom depth (m)	Number of individuals observed	Species
2016–08-25	3.5862	41.6697	Neuston sledge	300	0.5–1	704	3	*Aristeus antennatus*
2016–08-20	2.9417	41.5080	Neuston sledge	300	0.5–1	507	10	*Aristeus antennatus*
2017–02-19	2.7630	41.2000	Bongo	300	0–500	1,173	4	*Gennadas elegans*
2017–02-21	3.7199	41.2885	Bongo	300	0–618	2,401	3	*Gennadas elegans*
2017–02-17	2.8811	41.3418	Bongo	300	0–506	1,790	2	*Gennadas elegans*
2017–02-17	2.8811	41.3418	Bongo	300	0–506	1,790	2	*Gennadas elegans*

From the total of PZ I larvae caught in each sampling (527 in the summer and 11 in the winter), Carreton et al.^22^ performed extraction, amplification and sequencing of the Cytochrome Oxydase I (COI) and 16S rDNA molecular markers on randomly-selected individuals (24 in the summer and 4 in the winter). All summer individuals analysed were identified as *A. antennatus* and all winter individuals as *G. elegans.* The genetic distance values were 0.00 within each species and 0.15 between species, the latter calculated with 16S rDNA data. Carreton et al.^22^ also took Scanning Electron Microscopy (SEM) images and measurements of total length and carapace length for individuals of both taxa and sampling season.

### Drawings and measurements

Drawings and measurements were made following the methods and equipment presented by Bartilotti et al.^39^. Additionally, and since they are transparent, the larvae were stained with Chlorazol Black and Hematoxylin before being drawn. The long aesthetascs on the antennulae as well as the long plumose setae on the distal end of the exopods and on the uropods and telson were drawn truncated; the setules from setae were omitted from drawings when necessary. The drawings were then improved and digitally organized using GIMP software^40^. The observed individuals have been deposited at the Biological Reference Collection at the Institut de Ciències del Mar (ICM-CSIC) in Barcelona, Spain, under reference numbers ICMD002660 for *A. antennatus* and ICMD002661 for *G. elegans*.

## Data Availability

Data from this paper are available to readers upon request. The observed larvae have been deposited at the Biological Reference Collection at the Institut de Ciències del Mar (ICM-CSIC) in Barcelona, Spain, under reference numbers ICMD002660 for *A. antennatus* and ICMD002661 for *G. elegans*.
